# Characterization of Regulatory Dendritic Cells That Mitigate Acute Graft-versus-Host Disease in Older Mice Following Allogeneic Bone Marrow Transplantation

**DOI:** 10.1371/journal.pone.0075158

**Published:** 2013-09-10

**Authors:** Sabrina M. Scroggins, Alicia K. Olivier, David K. Meyerholz, Annette J. Schlueter

**Affiliations:** 1 Interdisciplinary Graduate Program in Immunology, University of Iowa Graduate College, Iowa City, Iowa, United States of America; 2 Department of Pathology, University of Iowa College of Medicine, Iowa City, Iowa, United States of America; Karolinska Institutet, Sweden

## Abstract

Despite improvements in human leukocyte antigen matching and pharmacologic prophylaxis, acute graft-versus-host disease (GVHD) is often a fatal complication following hematopoietic stem cell transplant (HSCT). Older HSCT recipients experience significantly increased morbidity and mortality compared to young recipients. Prophylaxis with syngeneic regulatory dendritic cells (DCreg) in young bone marrow transplanted (BMT) mice has been shown to decrease GVHD-associated mortality. To evaluate this approach in older BMT recipients, young (3–4 months) and older (14–18 months) DCreg were generated using GM-CSF, IL-10, and TGFβ. Analysis of young versus older DCreg following culture revealed no differences in phenotype. The efficacy of DCreg treatment in older BMT mice was evaluated in a BALB/c→C57Bl/6 model of GVHD; on day 2 post-BMT (d +2), mice received syngeneic, age-matched DCreg. Although older DCreg-treated BMT mice showed decreased morbidity and mortality compared to untreated BMT mice (all of which died), there was a small but significant decrease in the survival of older DCreg-treated BMT mice (75% survival) compared to young DCreg-treated BMT mice (90% survival). To investigate differences between dendritic cells (DC) in young and older DCreg-treated BMT mice that may play a role in DCreg function *in vivo*, DC phenotypes were assessed following DCreg adoptive transfer. Transferred DCreg identified in older DCreg-treated BMT mice at d +3 showed significantly lower expression of PD-L1 and PIR B compared to DCreg from young DCreg-treated BMT mice. In addition, donor DC identified in d +21 DCreg-treated BMT mice displayed increased inhibitory molecule and decreased co-stimulatory molecule expression compared to d +3, suggesting induction of a regulatory phenotype on the donor DC. In conclusion, these data indicate DCreg treatment is effective in the modulation of GVHD in older BMT recipients and provide evidence for inhibitory pathways that DCreg and donor DC may utilize to induce and maintain tolerance to GVHD.

## Introduction

Allogeneic hematopoietic stem cell transplant (HSCT) remains the only curative treatment modality for many hematopoietic disorders and malignancies. Acute graft-versus-host disease (GVHD) is an immune mediated disease whereby donor T cells are primed against recipient histocompatibility antigens resulting in expansion and differentiation of alloreactive T cells, the release of pro-inflammatory cytokines, and the recruitment of additional effector cell populations. This leads to damage of many tissues, most commonly skin, gastrointestinal tract, and liver. Despite improved human leukocyte antigen (HLA) matching and prophylaxis, GVHD remains a significant complication of HSCT. GVHD occurs in 20-40% of young and up to 70% of older allogeneic transplant recipients, and is fatal in approximately 30% of those patients [1,2]. In both humans and mice, older recipient age is an independent risk factor for increased incidence and severity of GVHD [3–7]. The number of older individuals undergoing HSCT is steadily growing [8], increasing the need for evaluation of therapies for GVHD specifically in older transplant recipients.

Induction of GVHD involves the priming of donor T cells by antigen presenting cell (APC) co-stimulation through CD28-CD80/86 and ICOS interactions; GVHD is inhibited by CTLA-4 and programmed death (PD)/programmed death ligand 1 (PD-L1) interactions [9–11]. Both residual recipient and donor APC are capable of inducing CD4 T cell mediated GVHD, while CD8 T cell mediated GVHD requires recipient APC for induction and donor APC to cross-present alloantigen to augment the response [12–15]. The increase in the allogeneic T cell response observed in older mice and accompanying increased severity of GVHD has been shown to be due to older recipient APC and their enhanced stimulation of donor T cells [6].

Although current pharmacological prophylactic and therapeutic approaches to interfere with T cell and/or APC function reduce severe GVHD, reciprocal increases in graft failure, relapse of malignant disease, infections, and viral-associated lymphoproliferative disorders limit their overall efficacy. Thus, many treatment regimens result in significant toxicities with no substantial increase in overall survival [16,17]. These shortfalls in current approaches to ameliorate GVHD, while preventing profound immunosuppression and maintaining the graft-versus tumor (GVT) response, underscore the need for improved therapeutic approaches that may be better provided by cellular therapies [16,18].

Dendritic cells (DC) are well accepted as the primary type of APC that contributes to adaptive immunity [19,20]. In addition to their conventional immunostimulatory role, DC also play a pivotal role in immune homeostasis and tolerance through their interactions with T cells and cytokine production [21]. In contrast to conventional DC (cDC), regulatory DC generated *in vitro* for therapeutic use (DCreg) (also referred to in the literature as alternatively activated DC or tolerogenic DC) express low levels of co-stimulatory markers, produce anti-inflammatory cytokines, induce regulatory T cell (Treg) development and promote T cell anergy [22–26]. In murine models, recipient-type DCreg were able to prevent lethal systemic inflammatory responses, allergic airway disease, solid organ allograft rejection, and lethal GVHD [22,27–30], and were an effective therapy for the treatment of allergic airway and autoimmune diseases [25]. Importantly, DCreg adoptive transfer in murine models of GVHD maintains the GVT response [22,30]. Although DCreg therapy ameliorates GVHD while maintaining GVT at least partially via Treg induction or expansion [22,30], the specific characteristics of DCreg that promote tolerance to alloantigens are currently not well understood.

The efficacy of DCreg therapy for GVHD has to our knowledge never been investigated in older bone marrow transplant (BMT) recipients. The purpose of the current study was to examine the therapeutic potential of DCreg adoptive transfer for the amelioration of GVHD in older mice relative to young mice, and secondarily, to begin to elucidate potential mechanism(s) by which DCreg mitigate GVHD. The results demonstrate that DCreg treatment is effective in alleviating GVHD in older BMT mice; however, these mice still experience mildly increased severity of GVHD compared to young DCreg-treated BMT mice, and slightly higher mortality. Phenotypic alterations in DC populations following DCreg adoptive transfer were evaluated to investigate differences between these cells in young and older BMT recipients that could contribute to the observed differences in morbidity and mortality. The inhibitory molecules PD-L1 and paired immunoglobulin receptor B (PIR B) were upregulated on young, but not older DCreg shortly following transfer relative to their expression prior to transfer. Lack of expression of either of these molecules on DCreg resulted in failure of their ability to ameliorate GVHD. In addition, donor-derived DC in both young and older DCreg-treated BMT mice upregulated expression of inhibitory molecules and downregulated expression of CD86 between d +3 and d +21 (3 and 21 d after BMT; all similar notation indicates number of days after BMT), indicating a less activated phenotype on donor-derived DC in mice surviving long-term. These novel results indicate that DCreg therapy can provide benefit to older as well as young BMT recipients at risk for GVHD, and begin to elucidate changes to DC populations *in vivo* following adoptive transfer of DCreg that may play an important role in the induction and maintenance of a GVHD-free state.

## Materials and Methods

### Ethics Statement

Animal care and procedures were carried out in accordance with The Association for Assessment and Accreditation of Laboratory Animal Care, International (AAA-LAC) and PHS Animal Welfare (A3021-01) mandates and approved by the University of Iowa Institutional Animal Care and Use Committee (Protocol No. 1111242). Accordingly, maximum efforts were made to minimize suffering.

### Mice

Recipient mice, young (3-5 mo) and older (14-18 mo) C57Bl/6 (B6, H-2K^b^) or BALB/c (H-2K^d^) mice were purchased from Harlan Sprague Dawley Inc. (Indianapolis, IN). Mice expressing GFP under the control of a beta-actin promoter [C57Bl/6-Tg (CAG-EGFP) 131Osb/LeySopJ] were purchased from The Jackson Laboratory (Bar Harbor, ME). PIR B-/- mice were a gift from Dr. Hiromi Kubagawa (University of Alabama School of Medicine, Birmingham, AL) [31]. PD-L1-/- mice were a gift from Dr. Randolph Noelle (Geisel School of Medicine, Dartmouth College, Hanover, NH) [32]. All mice were maintained in the specific pathogen-free Animal Care Facility at the University of Iowa.

### Flow Cytometric Reagents

Cells were stained with the following fluorochrome conjugated mAbs: MHC Class I (CL I) H-2K^b^ (AF6-88.5.5.3); CL I H-2K^d^ (SF1-1.1); MHC Class II (CL II) I-A/I-E (M5/114.15.2); CD11c (N418); CD40 (HM40-3); CD80 (16-10A1); CD86 (GL-1); PD-L1 (MIH5); PD-L2 (122); PIR B (326414); CCR9 (CD199; eBioCW-1.2); CD200R3 (Ba103); CD103 (2E7). Polyclonal purified rat immunoglobulin G (rIg; Jackson ImmunoResearch, West Grove, PA) was used as an isotype control. All cell samples were incubated with anti-CD16/32 (clone 2.4G2) and rat serum (Pel-Freez Biologicals, Rogers, AR) during staining to prevent background FcγR binding.

### DCreg and cDC Cultures

DCreg were prepared as previously described [22]. Briefly, bone marrow (BM) cells from BALB/c, B6, PIR B-/- or PD-L1-/- mice were cultured at 1 x 10^5^ cells/mL with human TGF-β1, and murine GM-CSF and IL-10 (Peprotech, Rocky Hill, NJ; 20 ng/ml each), for 8 d. LPS (Sigma-Aldrich, St. Louis, MO; 1 µg/ml) was added to the cultures for the last 24-48 h of culture. cDC were prepared similarly by culturing BM cells from BALB/c or B6 mice with murine GM-CSF and IL-4 (20 ng/ml each) followed by LPS activation.

### Flow Cytometric Staining and Analysis

Recipient splenocyte suspensions were centrifuged through FicoLite LM (Atlanta Biologicals, Lawrenceville, GA) and mononuclear cells were recovered from the interface. Non-adherent, cultured DCreg and cDC were recovered from culture and washed in balanced salt solution (BSS). Cell suspensions were stained with fluorochrome-conjugated or biotinylated Abs, followed by streptavidin-conjugated fluorochromes if necessary. The cells were then fixed with 0.1% formaldehyde for flow cytometric analysis.

Flow cytometric data were obtained on a Becton Dickinson LSR II or a Becton Dickinson FACSCanto II (San Jose, California) and analyzed using FlowJo software (TreeStar Inc, Ashland, OR). Dead cells were excluded by forward/orthogonal light scatter characteristics.

### ELISA

DCreg and cDC culture supernatants were harvested and stored at -80^o^C until analysis. Supernatants were assayed in triplicate using human/mouse TGFβ1, mouse IL-6, IL-10, IL-12p70, and TNFα Ready-Set-Go ELISA kits (eBioscience).

### GVHD Induction

Following a minimum of one week of acclimation in the animal care facility, mice received 11 Gray (Gy) (6 + 5 split dose 4 h apart; B6 mice) or 9.5 Gy (5 + 4.5 split dose 4 h apart, BALB/c mice) γ-irradiation with an 81-16A J.L. Shepherd Co (San Fernando, CA). irradiator equipped with a ^137^Cs source or X-irradiation from a Pantak Orthovoltage unit, Bipolar Series 2, HF-320 (Pantak Inc., East Haven, CT). Allogeneic BMT was performed by i.v. injection of complete MHC-mismatched BM cells (1.5 x 10^7^) and splenic mononuclear cells (5 x 10^7^ for B6 recipients; 5 x 10^6^ for BALB/c recipients) on day 0. DCreg treated recipients received 5 x 10^6^ recipient MHC-matched DCreg i.v. on day 2 post-BMT. As an indicator of morbidity, recipient mice were weighed daily.

### Histological Analysis

Following euthanasia, small bowel, colon, liver, and skin were prosected and fixed in 10% neutral buffered formalin for 48-96 hr. Tissues were then processed, paraffin-embedded, sectioned (4 µm), and stained with H & E for examination [33].

A previously published grading scale with modifications was used to assess histologic GVHD [34]. Blinded histological analyses were performed by evaluating and scoring processed tissues for histological severity using a scale of 0-4 as follows: 0- normal, 1- focal/mild, 2- diffuse/mild, 3- diffuse/moderate, 4- diffuse/severe. The tissues were evaluated on the following parameters: *Small bowel*-villous blunting, luminal sloughing/cell debris, crypt cell necrosis, crypt regeneration, crypt loss/destruction, lamina propria (LP) inflammation, and ulceration. *Colon*-colonocyte vacuolization, colonocyte attenuation, apoptosis, regeneration/proliferation, crypt destruction, LP inflammation, and mucosal epithelial inflammation. *Liver*-hepatocyte apoptosis, microabscess, hepatocyte mitosis, cholestasis, and steatosis. *Skin*-basal vacuolar damage, epidermal inflammation, epithelial/adnexal apoptosis, dermal infiltrate, cleft/microvesicle formation, separation of epidermis from dermis, acanthosis, hyperkeratosis, and pigmentary incontinence. A total score per organ was obtained by adding the score of each individual parameter for a specific organ.

### Statistical Analysis

Statistical significance was determined using a two-tailed Student *t* test or one-way ANOVA and Turkey-Kramer Multiple Comparisons test, where appropriate. The minimal level of confidence deemed statistically significant was *p* value <0.05.

## Results

DCreg treatment is effective in preventing GVHD mortality while maintaining GVT responses in young BMT mice [22,30]. As the need for HSCT in older patients is greatly increasing, the primary goal of this study was to evaluate the effectiveness of DCreg treatment in older (14-18 mo) BMT mice. Additionally, characteristics of older vs. young DC populations were compared both *in vitro* prior to transfer into BMT mice and *in vivo* following transfer, to begin to identify potential mechanisms attributable to DC by which protection from GVHD might be attained.

### Low co-stimulatory molecule expression on DCreg is accompanied by high expression of inhibitory molecules PIR B and PD-L1, and immunosuppressive cytokine production

**Figure 1:**
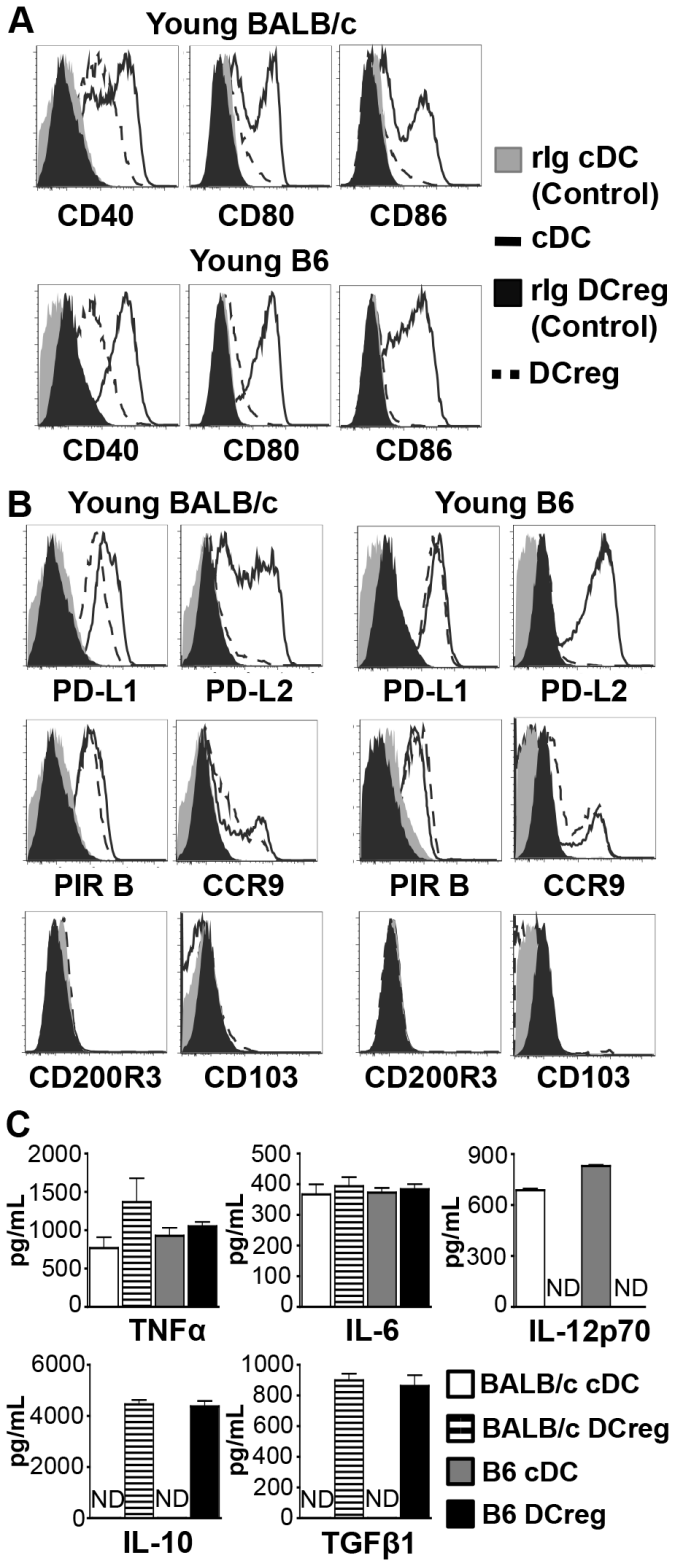
Regulatory dendritic cells express low levels of co-stimulatory molecules, but high levels of anti-inflammatory cytokines. Young BALB/c and B6 cDC and DCreg were stained for co-stimulatory (A) and inhibitory (B) cell surface molecules directly following culture. Live cells were gated and DC identified by gating on CD11c^+^ cells. (These cells were primarily CL II low.) rIg = rat IgG isotype control. Histograms are representative of N ≥ 4 independent experiments per group. (C) Cytokine concentrations in culture supernatants were measured by ELISA. ND, not detected. Data are mean ± SEM and represent 3 independent experiments.

While it is established that DCreg derived from BM of young mice *in vitro* express low levels of co-stimulatory molecules, the expression of inhibitory molecules is not well studied. The relative expression of co-stimulatory and inhibitory molecules may provide clues about potential mechanisms by which DCreg control GVHD. Therefore, co-stimulatory and inhibitory molecule expression was directly compared between cDC and DCreg from both B6 and BALB/c mice, at the end of culture. Confirming previous reports [22,35], young BALB/c and B6 DCreg expressed low levels of co-stimulatory molecules (CD40, CD80, and CD86) compared to cDC (Figure 1A). PIR B, PD-L1, PD-L2, CCR9, CD200R3, and CD103 have been reported on various DC populations *in vivo* under tolerizing conditions [36–41]. At the end of culture, neither cDC nor DCreg expressed CD200R3 or CD103, and expression of CCR9 was detected on a small fraction of both cDC and DCreg (Figure 1B). PIR B expression was detected on DCreg and cDC following culture. While PD-L2 was only expressed on cDC, PD-L1 was highly expressed on both cDC and DCreg. Since a low co-stimulatory: inhibitory molecule ratio on DC correlates with regulatory function [21], both BALB/c and B6 DCreg display a regulatory phenotype when compared with cDC.

Next, cytokine production by BALB/c and B6 cDC and DCreg was assessed in culture supernatants via ELISA. In both strains, cDC and DCreg produced comparable amounts of the inflammatory cytokines TNFα and IL-6, but only cDC produced IL-12p70. Similar to previous reports [24,25,27,29], DCreg but not cDC from both strains also produced significant levels of IL-10 and TGFβ1 (Figure 1C). This result was not due to residual IL-10 and TGFβ added at initiation of the culture, as DCreg washed and placed back into culture just prior to LPS stimulation maintained production of these immunosuppressive cytokines. No significant differences in cytokine levels were observed between washed and unwashed cultures (IL-10 p= 0.69, TGFβ1 p= 0.18). Taken together, these data demonstrate that at the end of culture, BALB/c and B6 DCreg express comparable low levels of co-stimulatory molecules and high levels of inhibitory molecules, and secrete immunosuppressive cytokines—evidence of DC with tolerogenic capacity.

### DCreg phenotype and cytokine production are comparable in young and older mice

**Figure 2:**
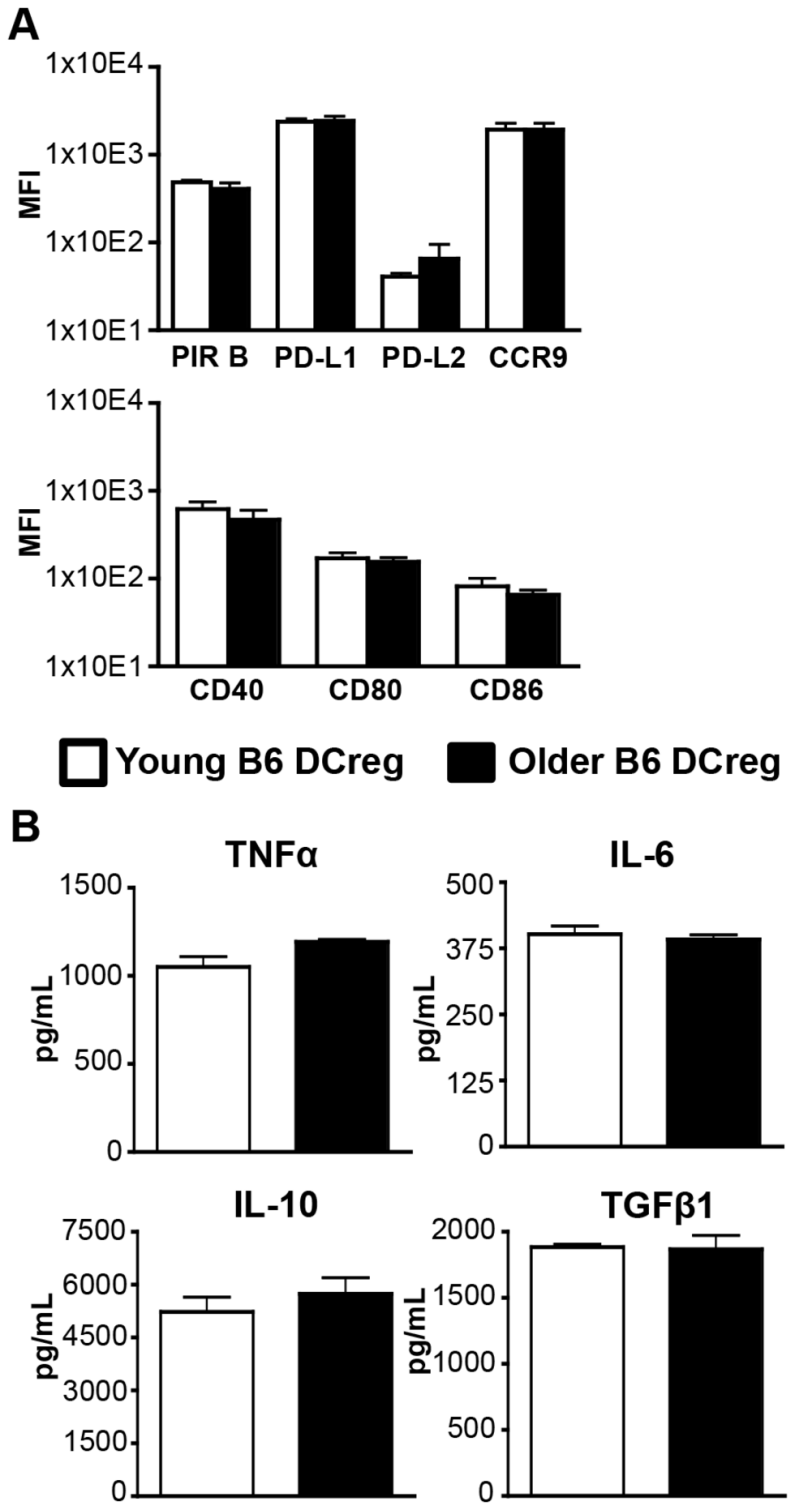
Expression of surface receptors and production of cytokines is comparable between young and older DCreg. DC were identified as in Figure 1. (A) Mean fluorescence intensity (MFI) of cell surface molecules on young and older B6 DCreg following culture. Data are mean ± SEM. N = 4-5 mice/group. (B) Young and older B6 DCreg culture supernatants were assessed for production of cytokines by ELISA. Data are mean ± SEM and represent 3 independent experiments.

To begin to assess whether older DCreg may also be effective at alleviating GVHD in older mice, young and older DCreg were first compared phenotypically and functionally *in vitro*. The age of the older mouse cohort was selected to be 14-18 mo to correlate with previous descriptions of outcomes in older BMT mice [6] and approximate corresponding ages at which older patients receive HSCT. Co-stimulatory and inhibitory molecule expression, as well as cytokine production was compared between DCreg generated from young and older B6 mice. Young and older DCreg expressed similar levels of co-stimulatory and inhibitory markers (Figure 2A) and there were no significant differences in inflammatory or immunosuppressive cytokine production (Figure 2B). These data are the first characterization of older DCreg and demonstrate that older DCreg have a similar phenotype and cytokine profile compared to young DCreg, supporting the potential for DCreg treatment in older BMT recipients.

### Acute GVHD induced in young and older B6 mice is ameliorated with DCreg treatment

**Figure 3:**
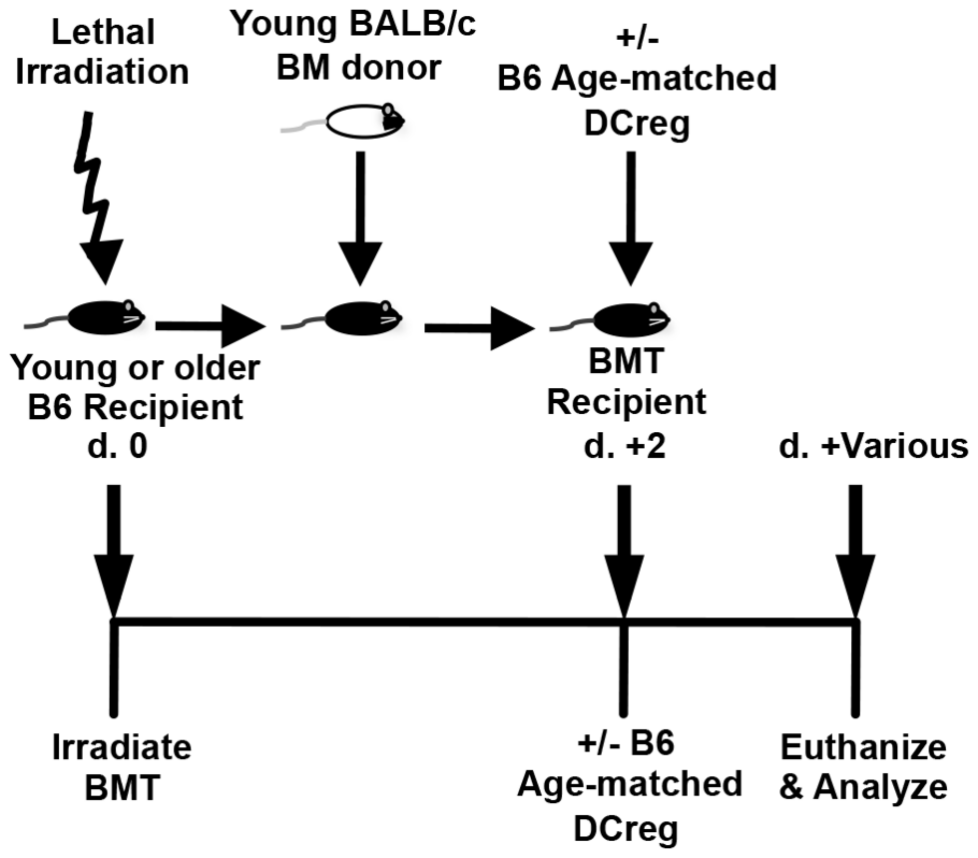
Acute GVHD induction in B6 mice. On d 0, lethally irradiated B6 mice received 5x10^7^ splenocytes and 1.5x10^7^ BM cells (BMT) from young BALB/c mice. 5x10^6^ age-matched DCreg were injected i.v. on d +2 and mice were subsequently euthanized at various time points for analysis.

**Figure 4:**
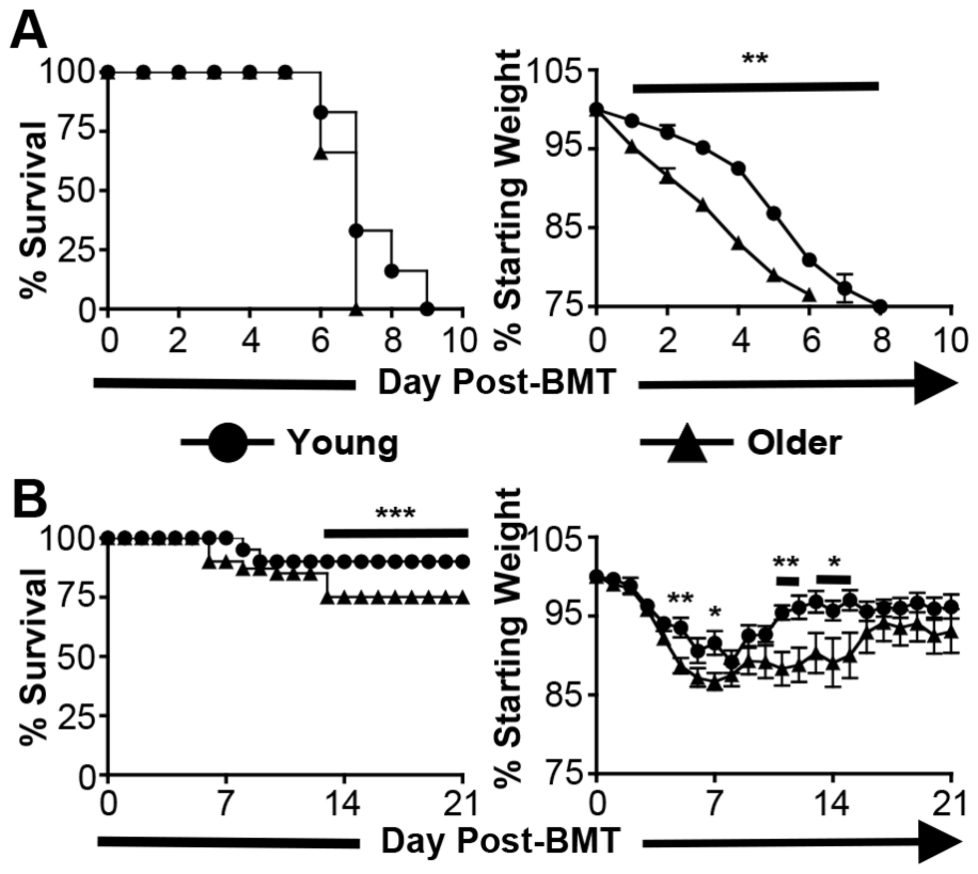
DCreg treatment attenuates GVHD in young and older BMT mice. (A) Young and older survival and morbidity following BMT in B6 mice. Data are ± SEM. N = 3-5 mice/group. (B) Survival and morbidity of young and older BMT mice following DCreg treatment. Data are mean ± SEM. N ≥ 16 mice/group. * = p<0.05, ** = p<0.01, *** = p<0.001.

To allow for the future investigation of additional potential mechanisms of DCreg function utilizing genetically deficient (knockout) mice on the B6 background, a B6 model of acute GVHD in young and older mice was established. On d 0, lethally irradiated B6 mice received 5x10^7^ splenocytes and 1.5x10^7^ BM cells from young BALB/c mice (Figure 3). Doses of less than 5x10^7^ splenocytes did not result in lethal GVHD or consistent elimination of recipient hematopoiesis (data not shown). Young B6 mice receiving this treatment (hereafter referred to as BMT mice) succumbed to GVHD by d +9, while older B6 BMT mice succumbed by d +7 (Figure 4A, left panel). Compared to young BMT mice, older BMT mice experienced significantly more weight loss from d +1 to d +6 (Figure 4A, right panel).

DCreg treatment was then administered to groups of young and older BMT mice. In DCreg-treated groups, 5x10^6^ age-matched recipient-type DCreg were injected i.v. on d +2 (Figure 3). Only DCreg from the recipient strain are effective in the prevention of murine GVHD [22]. Thus, to be clinically relevant, DCreg utilized in murine models must be age-matched as DCreg utilized to treat human HSCT recipients would need to be generated from those recipients. DCreg treatment in young and older BMT mice allowed survival rates of 90% and 75%, respectively, and decreased morbidity in both groups compared to non-DCreg treated BMT mice. However, older DCreg-treated BMT mice experienced significantly more weight loss between d +4 and d +15 compared to their young counterparts (Figure 4B). All mice surviving beyond d +15, regardless of age, maintained a healthy weight and no longer exhibited signs of GVHD (hunched posture, fur ruffling, diarrhea, lethargy) (Figure 4B, right panel and data not shown). DCreg therapy can also be used in older BMT mice to increase survival and decrease GVHD-associated morbidity; however, older DCreg-treated BMT mice experience slightly (but significantly) greater mortality and weight loss compared to young DCreg-treated BMT mice. This is the first report demonstrating DCreg therapy leads to greatly diminished short-term morbidity, as well as a lack of long-term morbidity in young and older BMT mice.

For comparison with previously published results, lethally irradiated young BALB/c mice were transplanted with B6 BM and splenocytes, and treated with BALB/c DCreg [22]. BALB/c DCreg-treated BMT mice had peak weight loss by d +7 and recovered by d +12 with an overall survival of 80% (Figure S1). Although young BALB/c DCreg-treated BMT mice lost a significantly greater percent of their baseline weight on d +4 to +7 compared to young B6 DCreg-treated BMT mice (Figure S1 vs. Figure 4B, right panels, p<0.01), there was no significant difference in overall survival (Figure S1 vs. Figure 4B, left panels; p=0.62). These data indicate that DCreg treatment of young B6 BMT mice results in at least as much improvement in morbidity and mortality as in young BALB/c BMT mice.

DCreg cultures contain both CD11c^+^ and double negative (DN; CD11c^-^ CL II^-^) populations (Figure S2A). Similar to CD11c^+^ DCreg, the DN population was positive for 33D1, CCR9 partial positive, and negative for CD40/80/86, CD103, CD200R3, as well as T, B, and NK cell markers: CD4, CD8, CD25, B220, and NK1.1. PD-L1 expression was detectable on DN DCreg, however, at much lower levels than on CD11c^+^ DCreg. Both populations of DCreg were also negative for other markers found on DC subsets: CD205 and CD207. Both the CD11c^+^ and DN DCreg populations expressed CD11b; therefore, the expression of additional molecules associated with myeloid derived suppressor cells (MDSC) [42] was examined. Ly6G, Ly6C, F4/80, IL-4Rα, and CD115 were negative on both populations (data not shown). Thus, neither population is composed of MDSC. The DN and CD11c^+^ populations in DCreg cultures may possess different capacities to confer protection from GVHD, and the experiments shown in Figure 4b utilized transfer of the combined populations recovered from the DCreg cultures. To evaluate this possibility, sort purified CD11c^+^ or DN populations from DCreg cultures were injected into young BMT mice on d +2. There was no difference in long-term survival of BMT mice that received CD11c^+^ vs. DN DCreg, indicating that both populations possess equivalent capacity to ameliorate GVHD (Figure S2B).

**Figure 5:**
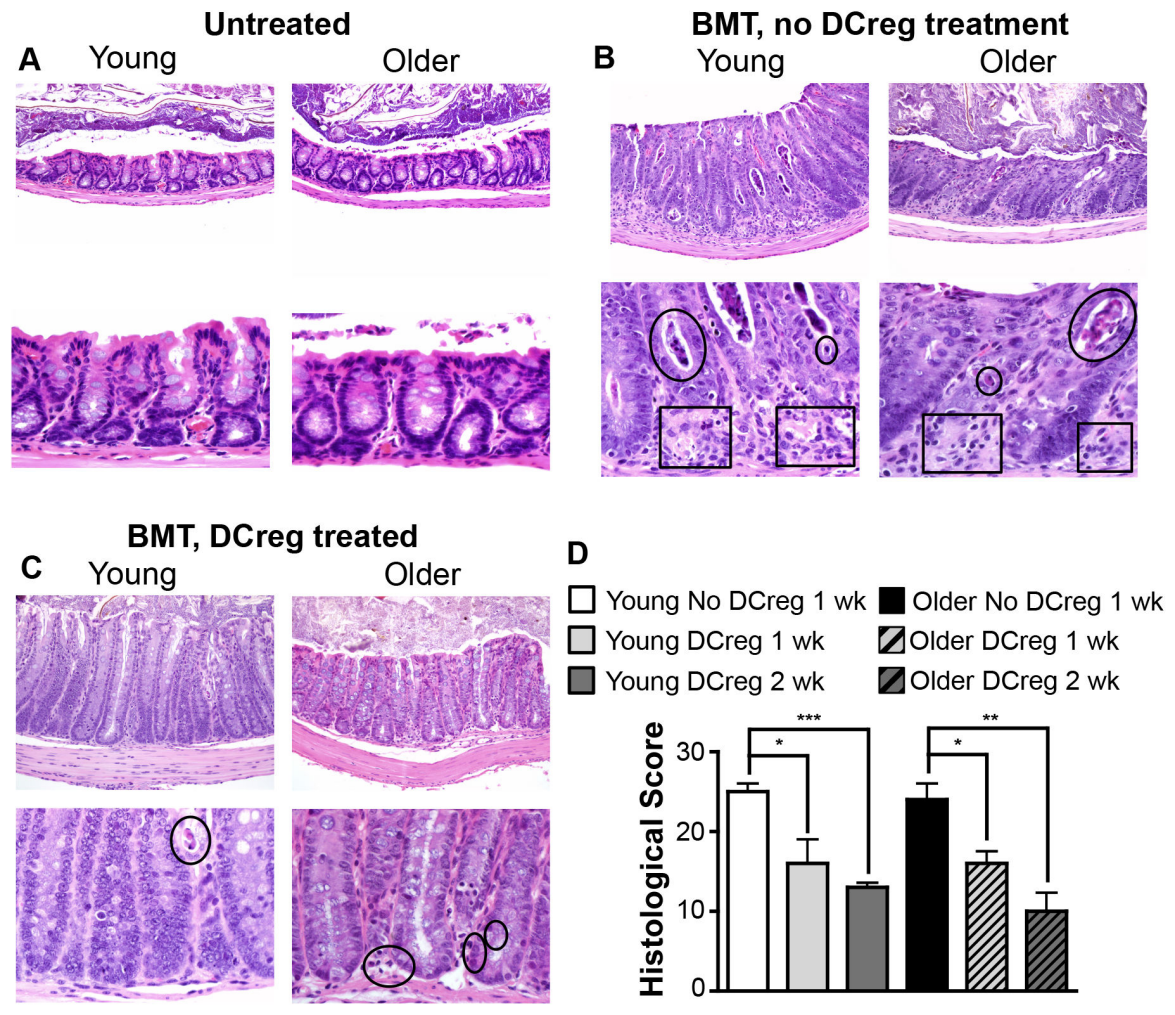
DCreg-treated BMT mice show reduced histologic evidence of GVHD. Young and older B6 mice were treated as described in Figure 3. Colon sections from (A) untreated B6 mice, (B) BMT mice, and (C) DCreg-treated BMT mice were stained with H & E. Original magnification was 20X (top panels) and 60X (lower panels). Tissues in (B) and (C) were obtained 1 wk post-BMT. Circles, necrosis/apoptotic bodies/cellular debris. Boxes, crypt degeneration/increased interstitial space. Representative images of 4-8 mice/group. (D) Total small intestine and colon histology scores of BMT mice 1 wk post-BMT, and DCreg-treated BMT mice 1 and 2 wk post-BMT were determined by adding individual organ scores as described in Methods. There were no statistical differences between young and older groups that received the same treatment. Data are mean ± SEM. N = 3-4 mice/group. * = p<0.05, ** = p<0.01, *** = p<0.001.

To address whether histological as well as clinical evidence of GVHD is proportionately reduced in young and older DCreg-treated BMT mice, small bowel, colon, liver, and skin were evaluated from BMT mice with and without DCreg treatment. Colons of young and older BMT mice demonstrated significant degeneration and loss of crypts, accompanied by evident apoptotic bodies, inflammation and replacement with connective tissue (Figure 5B; untreated colon is shown for comparison in Figure 5A). Furthermore, active proliferation of epithelial cells resulted in a lack of normal goblet cell abundance. These findings confirm that our BALB/c→B6 model does indeed represent acute GVHD. Comparatively, DCreg-treated BMT mice had less cellular debris and necrosis with relative preservation of crypt depth and goblet cell frequency. While young DCreg-treated BMT mice had a near absence of inflammation and apoptotic bodies, older DCreg-treated BMT mice had mild evidence of these changes (Figure 5C). Both young and older DCreg-treated BMT mice had a significant reduction in gut histological score (combined scores of small intestine and colon) by week 1 post-BMT that was further decreased by week 2 post-BMT (Figure 5D). However, the scores were not significantly different between young and older DCreg treated BMT mice at any time point. Liver and skin had minimal histological evidence of GVHD (total histology scores of 2-3 per tissue). In addition, young and older DCreg-treated BMT mice that survived long-term had no clinical or histological evidence of GVHD (data not shown and Figure S3). Together, these data confirm that DCreg treatment prevents severe GVHD shortly following BMT, and facilitates long-term disease-free survival in young and older BMT mice.

### Surface receptor expression is altered on DC from older vs. young DCreg-treated BMT mice in vivo

**Figure 6:**
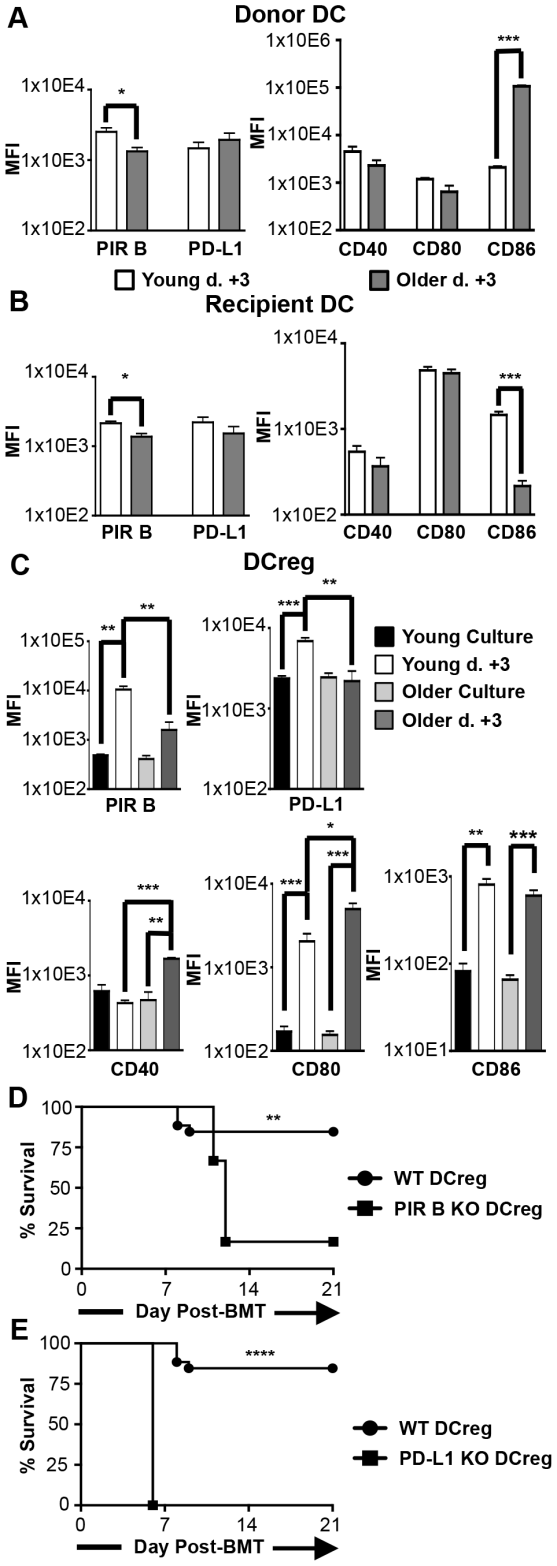
Dendritic cell surface receptor expression and function in DCreg-treated BMT mice. Young and older BMT mice were treated with GFP^+^ DCreg. DC populations were gated as described in Figure S4 to distinguish between donor DC (H-2K^d+^), recipient DC (GFP- H-2K^b+^), and DCreg (GFP^+^ H-2K^b+^). Co-stimulatory and inhibitory molecule expression on (A) donor DC, (B) recipient DC, and (C) DCreg on d +3 are compared to expression at the end of culture. Data are mean ± SEM. Young BMT mice were treated with PIR B-/- (D) or PD-L1-/- (E) DCreg and survival monitored for 3 wks. N > 4 mice/group and represent two independent experiments each for A-C, D, and E. * = p<0.05, ** = p<0.01, *** = p<0.001, **** = p<0.0001.

Both young and older DCreg-treated BMT mice demonstrated greatly improved survival and diminished morbidity relative to age-matched BMT mice that did not receive DCreg. However, significantly decreased survival and increased morbidity was nevertheless observed in older vs. young DCreg-treated BMT mice. The latter findings may be due to differences in one or more of the DC populations present in young vs. older mice: transferred DCreg, residual recipient DC, or donor-derived DC. To examine this possibility, young and older BMT mice were treated with GFP^+^ B6 DCreg as in Figure 3, and DC populations were identified and analyzed for co-stimulatory and inhibitory molecule expression on d +3. (Cytokine production was not analyzed due to the low numbers of DC recoverable from mice shortly after BMT.) The DC populations were identified as H-2K^d+^ (donor), H-2K^b+^ GFP^-^ (recipient), and H-2K^b+^ GFP^+^ (DCreg) (Figure S4). Evaluation of these DC populations in young DCreg-treated BMT mice at d +3 demonstrated that expression of the inhibitory molecule PIR B was significantly increased on donor and residual recipient DC compared to their older counterparts (Figure 6A and B, left panel). Evaluation of co-stimulatory molecule expression on these same populations demonstrated that donor DC from older DCreg-treated BMT mice expressed higher levels of CD86 than those from young DCreg-treated BMT mice (Figure 6A, right panel). Surprisingly, CD86 was elevated on young relative to older recipient DC (Figure 6B, right panel). It is unclear what role, if any, this difference plays in the somewhat poorer survival of older DCreg treated BMT mice. Overall, donor and recipient DC populations display more robust inhibitory molecule expression in young than in older mice following DCreg treatment. When the relative expression of co-stimulatory molecules and inhibitory molecules is compared on donor DC, the overall phenotype suggests a more immunosuppressive population in young DCreg-treated BMT mice than in similarly treated older mice.

Transferred DCreg identified in spleens of young DCreg-treated BMT mice expressed higher levels of both PIR B and PD-L1 than DCreg identified in spleens of older DCreg-treated BMT mice (Figure 6C, top right panel). Comparison of DCreg directly following culture to those from d +3 DCreg-treated BMT mice revealed a significant upregulation of these inhibitory molecules on young but not older DCreg following adoptive transfer (Figure 6C, top panel). Comparison of co-stimulatory molecule expression on DCreg isolated at d +3 showed significantly higher levels of CD40 and CD80 on older vs. young DCreg (Figure 6C, lower panel). The increased expression was induced *in vivo*, as no difference in young vs. older DCreg CD40 or CD80 levels was observed at the end of culture (Figure 2A). CD80 and CD86 increased on DCreg in both young and older mice following adoptive transfer, while CD40 only increased on older DCreg.

DCreg were detectable in the spleen of young and older GFP+ DCreg-treated mice by flow cytometry at d +3. Concordantly, DCreg were also detected by immunohistochemistry (IHC) in the intestines of these mice. DCreg migrated to the small intestine (Figure S5) and colon (Figure S6) in young mice, but were only found in the small intestine of older mice (present at both d +3 and d +5). DCreg were not identified at either time point in the colon of older DCreg-treated BMT mice (data not shown).

In summary, comparison of young and older DCreg before and shortly after transfer into BMT mice demonstrates that older DCreg failed to increase inhibitory molecule expression while significantly upregulating co-stimulatory molecule expression following adoptive transfer, while young DCreg upregulated inhibitory molecules as well as co-stimulatory molecules. Adoptively transferred DCreg are able to traffic to both the small intestine and colon (major targets of GVHD) of young BMT mice, but apparently only the small intestine of older DCreg-treated BMT mice. These data demonstrate for the first time that phenotypic differences rapidly develop between young and older DCreg following transfer into BMT mice, and these cells quickly traffic to GVHD target organs. However, DCreg in young BMT mice successfully migrated to both the small intestine and colon while DCreg in older BMT mice were only observed in the small intestine. These differences provide a potential mechanism for modestly increased morbidity and mortality in older DCreg-treated BMT mice relative to their young counterparts.

### PIR B and PD-L1 expression on DCreg is required for DCreg-mediated amelioration of GVHD

The upregulation of PIR B and PD-L1 on DCreg *in vivo* following adoptive transfer suggested that these molecules may play a role in DCreg-mediated amelioration of GVHD. To investigate the requirement for PIR B or PD-L1 on DCreg following BMT, DCreg were generated from young wild type (WT), PIR B-/- or PD-L1-/- mice for adoptive transfer on d +2. BMT mice treated with PIR B-/- or PD-L1-/- DCreg had a significant reduction in overall survival compared to BMT mice treated with WT DCreg (Figure 6D,E). In fact, BMT mice treated with PD-L1-/- DCreg all succumbed to GVHD by d +6. Due to skin ulcerations, PIR B-/- and PD-L1-/- mice were unable to be maintained past 10 mo of age. Therefore the requirement for PIR B or PD-L1 on older DCreg *in vivo* could not be evaluated. Taken together, the data in Figure 6C–E indicate that PIR B and PD-L1 are quickly upregulated on DCreg following adoptive transfer into young BMT mice but not older BMT mice; and their expression by DCreg is required for DCreg-mediated tolerance to alloantigens (at least in young mice).

### DCreg-treated BMT mice upregulate inhibitory molecule expression on donor DC at d +21

**Figure 7:**
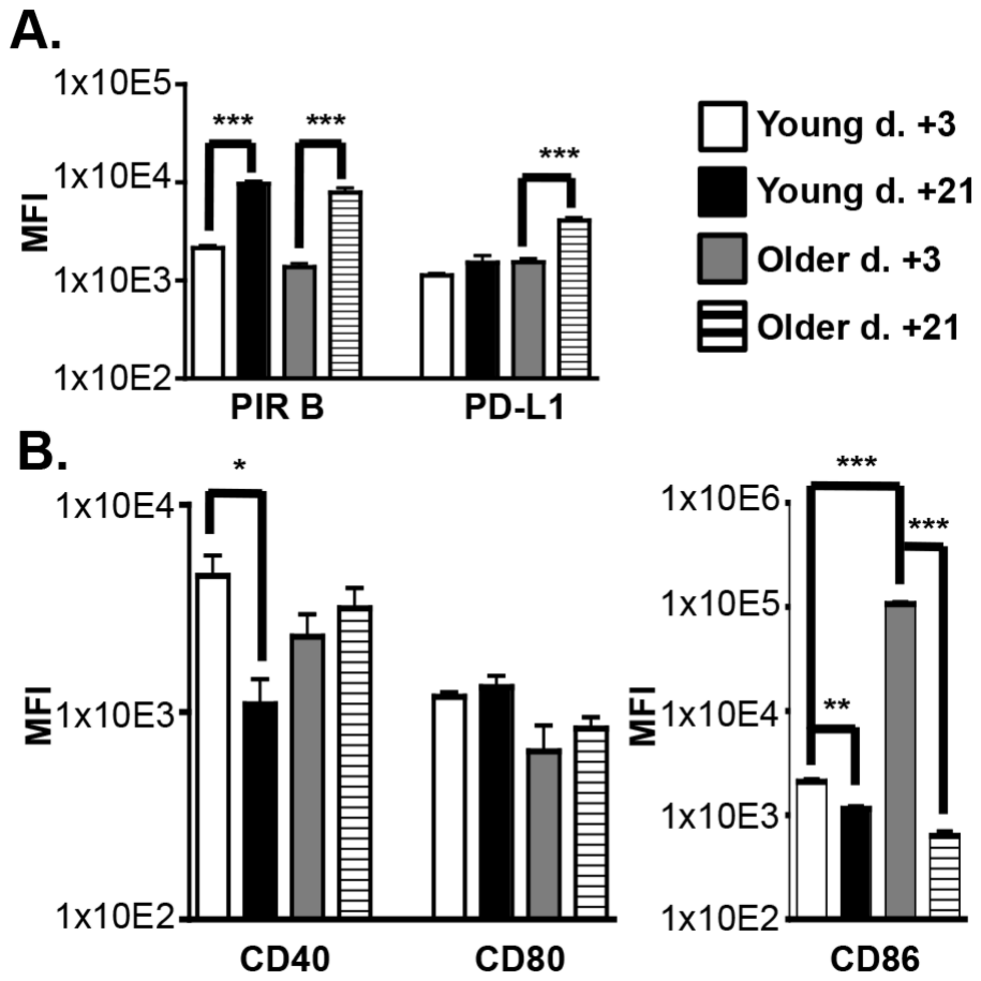
Co-stimulatory and inhibitory molecule expression is altered on donor DC from young and old DCreg-treated BMT mice at d +3 vs. d +21. Young and older BMT mice were treated with GFP^+^ DCreg. DC populations were gated as described in Figure S4 identify donor DC (H-2K^d+^) expression of (A) inhibitory and (B) co-stimulatory molecules. Data are mean ± SEM. N = 4-5 mice/group and represent two independent experiments. * = p<0.05, ** = p<0.01, *** = p<0.001.

Because transferred DCreg would be expected to only transiently survive in BMT mice, it seemed unlikely that this population alone could account for long-term GVHD-free survival. However, donor DC could still play a significant role in maintaining tolerance to allogeneic hematopoiesis. To begin to evaluate this possibility, DC co-stimulatory and inhibitory molecule expression was evaluated at d +21 for comparison with d +3. As anticipated, recipient DC and DCreg were no longer detectable at d +21 (data not shown). Interestingly, both young and older mice that recovered from mild GVHD and survived to d +21 had generally upregulated inhibitory molecule and downregulated co-stimulatory molecule expression on donor DC (Figure 7A and 7B). Specifically, in young DCreg-treated BMT mice, PIR B was significantly upregulated while CD40 and CD86 were significantly downregulated at d +21 relative to d +3. In older DCreg-treated BMT mice, both PIR B and PD-L1 were significantly increased on donor DC, while only CD86 expression was downregulated by d +21. These data elucidate additional inhibitory mechanisms (PIR B and PD-L1) that donor DC may utilize to maintain protection from GVHD in young and older DCreg-treated BMT mice, and indicate that the mechanisms may differ with age of the recipient.

## Discussion

DCreg have shown great potential in the treatment and/or prevention of a variety of diseases, including allergic and autoimmune diseases, as well as lethal GVHD. Studies utilizing DCreg have focused on young mice as the source of DCreg, and infusion into young recipients [22,25,27–30,43]. It is clear that GVHD is more prevalent and presents with increased severity in older HSCT recipients [3–7], underscoring the need for an effective therapy to prevent and treat GVHD in an older recipient population. This study is the first to characterize and evaluate older DCreg generation and demonstrate application in the amelioration of GVHD in older BMT recipient mice. It is also the first to investigate changes in DC populations *in vivo* (including DCreg) following DCreg treatment for the control of GVHD.

Prior to *in vivo* transfer, cultured young and older DCreg express very low levels of co-stimulatory molecules and high levels of inhibitory molecules (Figures 1 and 2). These data are consistent with reports that low co-stimulatory: inhibitory molecule ratios are present on tolerogenic DC [21]. IL-10 and TGFβare immunosuppressive cytokines involved in Treg function; TGFβis also critical in the development and maintenance of Treg [44,45]. In accordance with previous reports describing DC with regulatory functions [24,25,27,29], *in vitro* generated DCreg produced substantial amounts of IL-10 and TGFβ (Figure 1C and Figure 2B). In addition, DCreg secreted undetectable/minimal amounts of the inflammatory cytokine IL-12, but produced comparable amounts of IL-6 and TNFα as cDC. The latter finding is in contrast to previous reports [24,25,27] in which IL-6 and TNFα production by DCreg was much less than from cDC, and may be due to differences in culture conditions. However, the production of these cytokines did not prevent amelioration of GVHD *in vivo*.

When BMT mice were treated with age-matched young or older B6 DCreg, GVHD was largely prevented and survival was markedly enhanced relative to untreated BMT mice, but young mice still had slightly less morbidity and increased survival compared to older mice (Figures 4B and 5). DCreg-treated BMT mice also had reduced colon GVHD histological scores compared to untreated BMT mice, that continued to decrease with increasing time post-BMT (Figure 5D). GVHD was essentially undetectable in BMT mice that survived to d +125 (Figure S3). Importantly, these data demonstrate that DCreg may be used as an effective therapy in the mitigation of GVHD in older BMT mice, while highlighting the need for a better understanding of GVHD induction and DCreg function in young vs. older recipients.

Several reports provide *in vitro* evidence that DCreg suppress T cell responses and induce Treg in an antigen-dependent manner [26,30,35]. However, the characteristics of transferred DCreg that facilitate T cell suppression and Treg induction in the BMT setting are currently unclear. This study aims to identify what mechanism(s) DCreg utilize to accomplish this result either directly, or by altering other DC populations *in vivo*. While the co-stimulatory molecule expression patterns on DCreg prior to adoptive transfer have been reported, no studies have evaluated inhibitory molecule expression on BM-derived *in vitro* generated DCreg. To this end, the expression of multiple inhibitory molecules on DCreg following culture and after adoptive transfer was investigated (Figure 6C). In contrast to a previous report [40], our DCreg did not express CD200R3 at the end of culture. This disparity may be attributed to differences in culture conditions and/or the use of different anti-CD200R3 clones. Importantly, Ba103 (anti-CD200R3) did stain basophils, confirming the antibody was working appropriately (data not shown). Tolerogenic plasmacytoid DC expressing CCR9 have been shown to suppress GVHD [36]. As only a small fraction of young and older DCreg expressed CCR9 (14-17%; Figure 1B and data not shown), it seems unlikely that CCR9 plays a major role in DCreg amelioration of GVHD. CD103 is expressed on populations of skin and gut-associated DC with regulatory capacity, as well as on Treg populations [46,47]. BM-derived DCreg generated *in vitro* do not express CD103 (Figure 1B and data not shown). This finding may reflect a requirement for site-specific interactions to induce CD103 expression on DC [48]. Overall, the phenotype of DCreg at the end of culture indicates that CCR9, CD200R3, and CD103 are unlikely to be important for DCreg function in the BALB/c→B6 or B6→BALB/c GVHD models.

PD-L1 and PD-L2 are type I transmembrane proteins that regulate immune activation and tolerance [49]. PD-L1 is widely expressed on hematopoietic cells and non-hematopoietic cells [50], while PD-L2 expression is restricted to DC, macrophages, B1 B cells, and mast cells. PD-L2 signaling through PD-1 has been characterized as an inhibitory interaction, but there is also evidence that PD-L2 expression on DC is important in activation of naïve T cells [49,51]. In concordance with previous reports of the downregulation of PD-L2 in the presence of IL-10 on BM-derived DC [52] and consistent with a potential activation function, cDC but not DCreg express high levels of PD-L2 (Figures 1B and 2). Therefore, DCreg appear not to use PD-L2 as a regulatory molecule to ameliorate GVHD.

PD-L1 has been shown to exert inhibitory signals through either PD-1 and/or CD80 and is capable of delivering reverse signaling into the PD-L1 expressing cell (including T cells and DC) to downregulate activation [37,49,53]. In GVHD models, overexpression of PD-L1 on DC inhibits allogeneic lymphocyte activation *in vitro*, and recipient APC enhance expansion and survival of donor-derived Treg via CD80/PD-L1 interactions in BMT mice [11,54]. In the current study, PD-L1 was highly expressed on young and older DCreg directly following culture (Figure 2). In addition, young DCreg upregulated PD-L1 expression following adoptive transfer at d +3 (Figure 6C) and young DCreg required PD-L1 to mitigate GVHD (Figure 6E). Finally, donor DC upregulated PD-L1 (and downregulated co-stimulatory molecules) in young and older DCreg-treated BMT mice at d +21 compared to d +3 (Figure 7), resulting in donor DC that have a regulatory phenotype. It is then reasonable to hypothesize that PD-L1 expressing DCreg induce PD-L1 on donor T cells and promote the generation, expansion, and survival of donor-derived Treg. These Treg could then go on to induce PD-L1 expression on donor-derived DC, resulting in their conversion to tolerogenic DC and perpetuation of tolerance to alloantigens.

PIR B expressed on APC delivers inhibitory signals by binding to CL I [38,41]. Treatment with PIR B transfected DC has been shown to prevent lethal GVHD, and PIR B deficiency in BMT recipient mice results in exacerbated GVHD [55,56]. Young and older DCreg express PIR B *in vitro* (Figure 2A), and PIR B expression at d +3 was significantly higher on young (but not older) DCreg compared to the phenotype observed at the end of culture (Figure 6C). Additionally, PIR B deficiency in DCreg resulted in reduced overall survival in young BMT recipient mice (Figure 6D), indicating it is required for optimal mitigation of GVHD by DCreg. These data, when considered with the lack of PD-L1 upregulation on older DCreg following adoptive transfer at d +3 suggest young and older DCreg-treated mice may utilize divergent mechanisms to prevent and maintain tolerance to GVHD. Additionally, alterations in DCreg upregulation of PIR B and PD-L1 expression (and downregulation of CD40 and CD80) may contribute to the small but significant differences in mortality and morbidity between young and older DCreg-treated mice.

In addition to a less tolerogenic phenotype of older DCreg *in vivo*, increased baseline severity of GVHD in older mice may contribute to the modestly reduced efficacy of DCreg treatment in older mice. The observation that older BMT mice had a faster onset of disease and increased morbidity compared to young BMT mice (Figure 4A) provides evidence for intrinsic differences between young and older HSCT recipients (apart from DCreg function). Given the increased disease severity in older mice at baseline, it may be more difficult for older DCreg to exert their inhibitory function in this environment. Finally, differences in pro-inflammatory or inhibitory cytokine production by young vs. older DCreg *in vivo* (which we were unable to evaluate) could contribute to slightly poorer tolerance induction by older DCreg.

In summary, this study demonstrates that DCreg therapy is a promising approach to alleviate GVHD in older BMT recipients. Although older DCreg-treated BMT mice do not fare quite as well as their young counterparts, 75% survival with minimal long-term morbidity is observed in these mice. Additional investigation into the factors contributing to the minor differences in survival and morbidity observed between young and older BMT recipients may provide for further optimization of DCreg treatment in older BMT mice. These differences are likely due to a combination of environmental factors present in older recipients following irradiation, as well as differential sensitivity of adoptively transferred DCreg to this environment. Further understanding of the mechanisms by which DCreg function in the control and prevention of GVHD is vital for continued development of this approach for therapeutic purposes in young and older humans.

## Supporting Information

Figure S1 **Young BALB/c DCreg-treated BMT mice survive with minimal clinical evidence of GVHD.** Young BALB/c mice were treated as described in Figure 3, except donor and recipient strains were reversed. Briefly, B6 bone marrow and splenocytes were transferred on d +0 and young BALB/c DCreg administered d +2. Mice were then monitored for survival and morbidity. Data are mean ± SEM. N = 10 mice/group.
(TIF)

Figure S2 **Comparable survival between young BMT mice treated with either double negative or CD11c^+^ DCreg.** (A) Young cDC and DCreg directly isolated from culture were stained for CD11c and CLII expression. Dashed line, double negative cells (DN, CD11c^-^ CL II^-^); Solid line, CD11c^+^ cells. Data are representative of >10 independent experiments. (B) Cells isolated from DCreg cultures were sorted based on DN or CD11c+ gates as designated in (A). Sort-purified cells (≥97% purity) were injected into young BMT mice on d +2 and followed for survival. N = 8 mice/group; 2 independent experiments.
(TIF)

Figure S3 **Surviving young and older B6 DCreg-treated mice lack evidence of GVHD at late time points.** H & E of colon sections from WT and DCreg-treated BMT mice at d +125. Original magnification was 20X (top panels) and 60X (lower panels).
(TIF)

Figure S4 **Gating strategy for identifying DC populations in DCreg-treated BMT mice.** Splenocytes from d +3GFP^+^ DCreg-treated mice were stained for H-2K^b^, H-2K^d^, CD11c, and CL II for identification of DC subsets. Donor DC are H-2K d+CD11c^+^CL II^+^; Recipient DC are H-2K b+GFP^-^CD11c^+^CL II^+^; transferred DCreg are H-2K b+GFP^+^. The relatively large CL II^+^ CD11c- population in the donor gate is likely composed of activated T and B cells.
(TIF)

Figure S5 **DCreg traffic to the small intestine, a GVHD target organ, following adoptive transfer.** Young and older B6 mice were treated as described in Figure 3, except GFP+ DCreg were utilized for adoptive transfer. Following euthanasia, small intestine was prosected, rolled into a coil, and fixed, processed and sectioned as described in Materials and Methods. Sections were stained with a rabbit polyclonal anti-GFP antibody (Rockland Immunochemicals Inc., Gilbertsville, PA or Abcam, Cambridge, MA). Small intestine from GFP+ DCreg-treated BMT mice and untreated small intestine from GFP transgenic mice were stained for positive controls (top photograph). DCreg were also identified in the spleens of both young and older DCreg-treated BMT mice at both d +3 and d +5 (positive control concordant with flow cytometric results; data not shown). Negative controls included tissues obtained from naïve B6 mice or BMT mice that did not receive GFP+ DCreg as well as intestinal tissues obtained from GFP transgenic mice stained with isotype control antibody alone (data not shown). Original magnification = 40X and 60X in the upper and lower panels respectively, in the pair of images from each mouse.
(TIF)

Figure S6 **DCreg traffic to the colon, a GVHD target organ, following adoptive transfer.** Young and older B6 mice were treated as described in Figure 3, except GFP+ DCreg were utilized for adoptive transfer. Following euthanasia, small intestine and colon were prosected, rolled into a coil, and fixed, processed and sectioned as described in Materials and Methods. Sections were stained with a rabbit polyclonal anti-GFP antibody (Rockland Immunochemicals Inc., Gilbertsville, PA or Abcam, Cambridge, MA). Colon from GFP+ DCreg-treated BMT mice and untreated colon from GFP transgenic mice were stained for positive controls (top photograph). DCreg were also identified in the spleens of both young and older DCreg-treated BMT mice at both d +3 and d +5 (positive control concordant with flow cytometric results; data not shown). Negative controls included tissues obtained from naïve B6 mice or BMT mice that did not receive GFP+ DCreg as well as intestinal tissues obtained from GFP transgenic mice stained with isotype control antibody alone (data not shown). Original magnification = 40X and 60X in the upper and lower panels respectively, in the pair of images from each mouse.
(TIF)
