# Supplementary figures and images for: Characterization of Regulatory Dendritic Cells That Mitigate Acute Graft-versus-Host Disease in Older Mice Following Allogeneic Bone Marrow Transplantation
Source: PLoS One. 2013 Sep 10;8(9):e75158. doi: 10.1371/journal.pone.0075158 (PMC3769303; doi:10.1371/journal.pone.0075158)

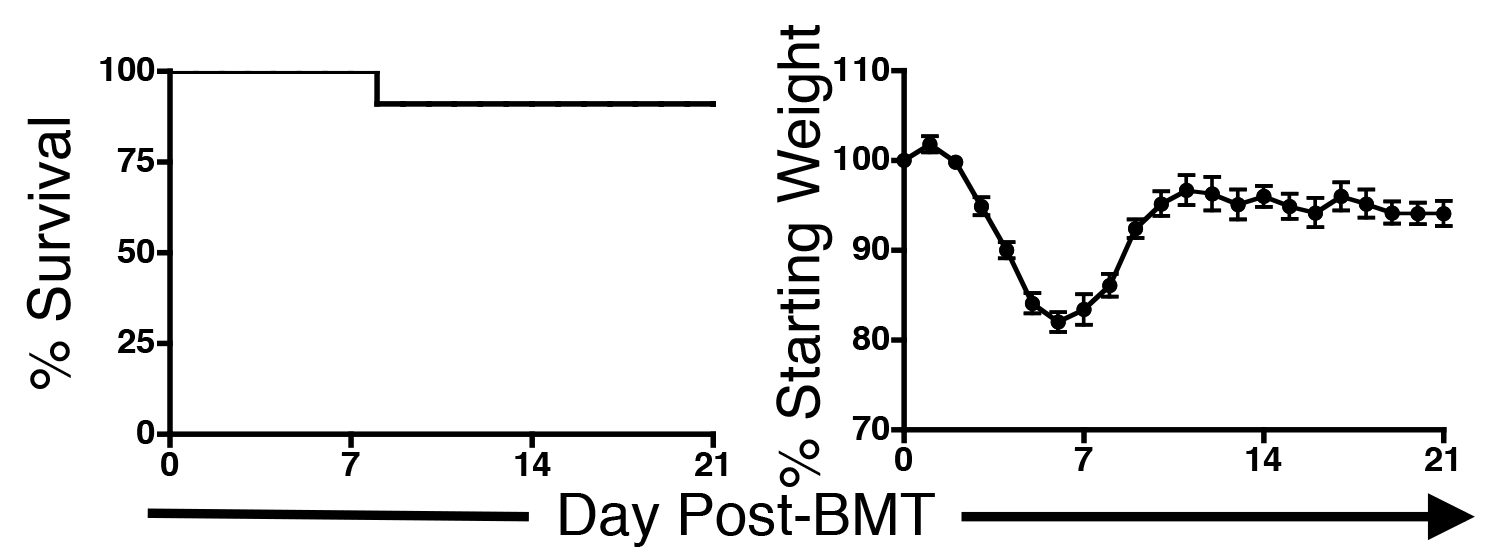

Supplement: Figure S1 — Young BALB/c DCreg-treated BMT mice survive with minimal clinical evidence of GVHD. Young BALB/c mice were treated as described in Figure 3, except donor and recipient strains were reversed. Briefly, B6 bone marrow and splenocytes were transferred on d +0 and young BALB/c DCreg administered d +2. Mice were then monitored for survival and morbidity. Data are mean ± SEM. N = 10 mice/group. (TIF) [file pone.0075158.s001.tif]

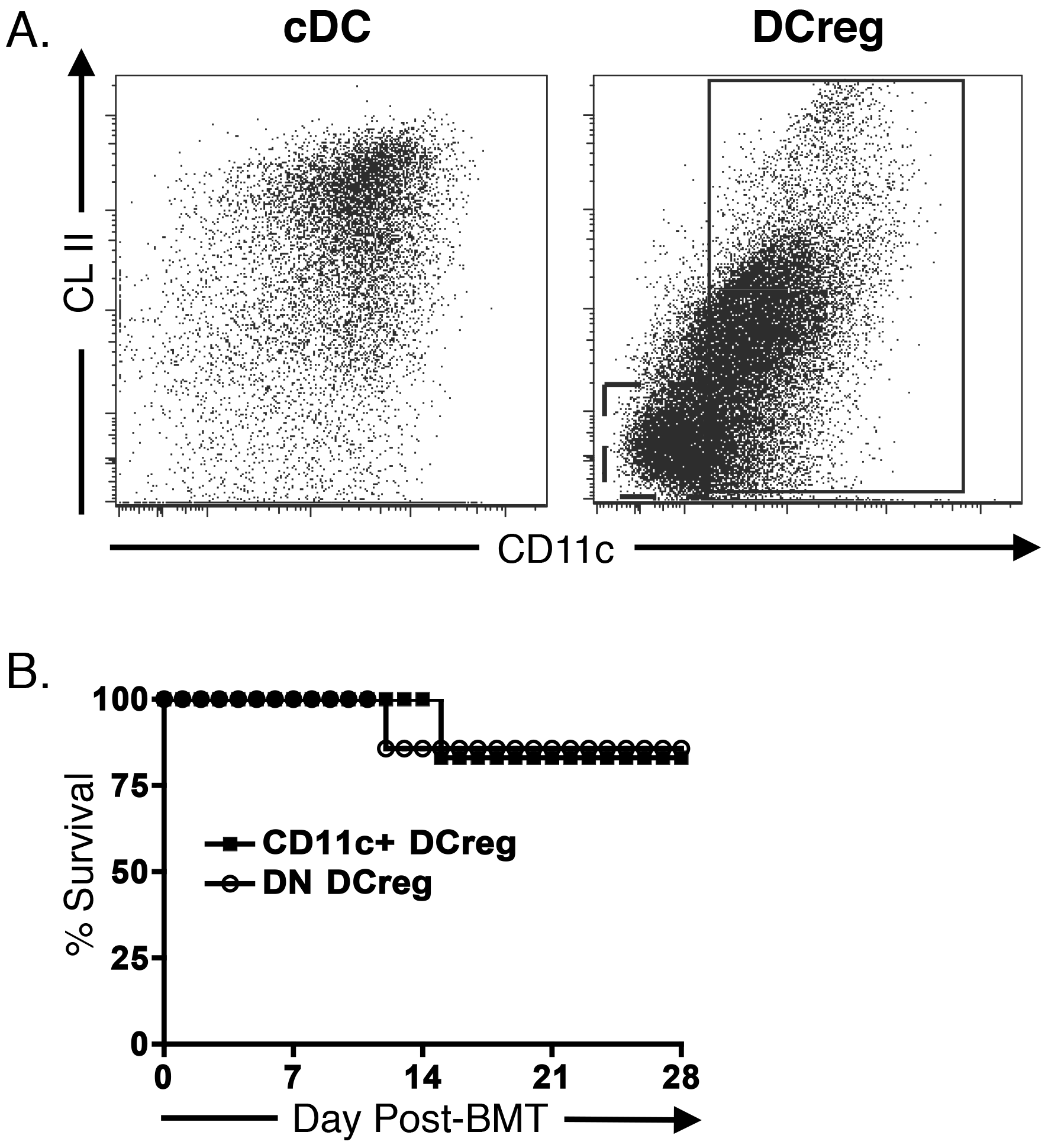

Supplement: Figure S2 — Comparable survival between young BMT mice treated with either double negative or CD11c+ DCreg. (A) Young cDC and DCreg directly isolated from culture were stained for CD11c and CLII expression. Dashed line, double negative cells (DN, CD11c- CL II-); Solid line, CD11c+ cells. Data are representative of >10 independent experiments. (B) Cells isolated from DCreg cultures were sorted based on DN or CD11c+ gates as designated in (A). Sort-purified cells (≥97% purity) were injected into young BMT mice on d +2 and followed for survival. N = 8 mice/group; 2 independent experiments. (TIF) [file pone.0075158.s002.tif]

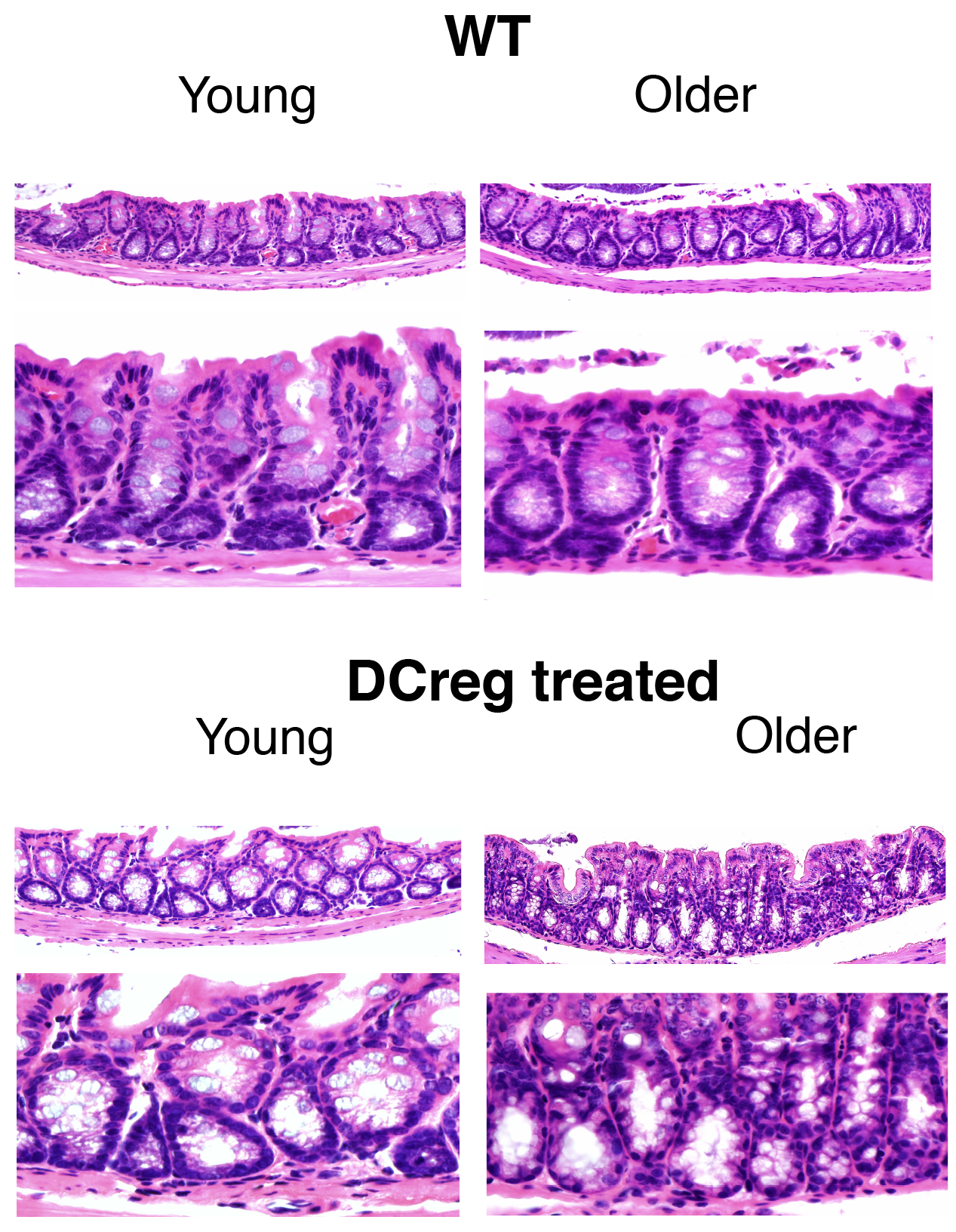

Supplement: Figure S3 — Surviving young and older B6 DCreg-treated mice lack evidence of GVHD at late time points. H & E of colon sections from WT and DCreg-treated BMT mice at d +125. Original magnification was 20X (top panels) and 60X (lower panels). (TIF) [file pone.0075158.s003.tif]

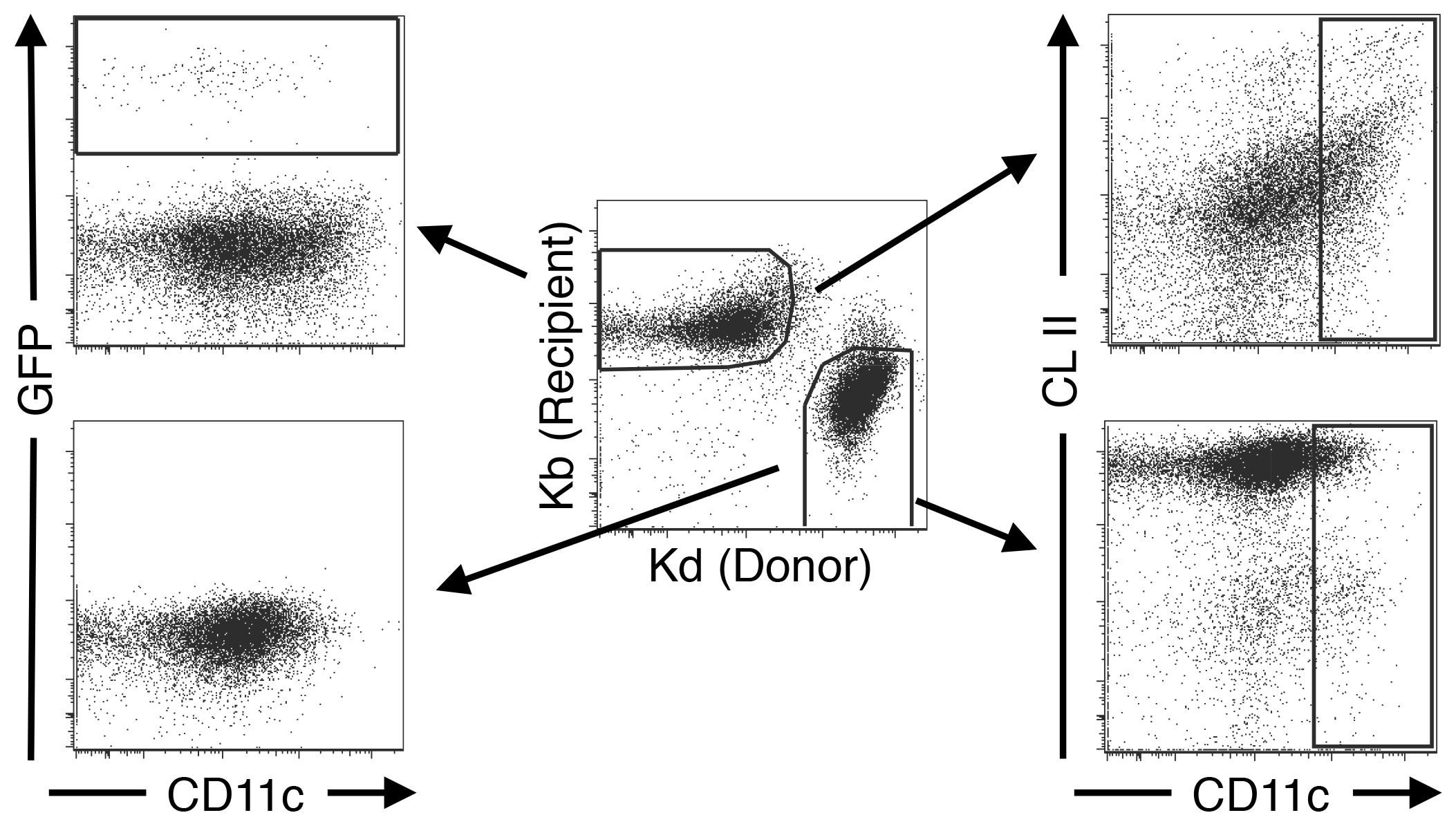

Supplement: Figure S4 — Gating strategy for identifying DC populations in DCreg-treated BMT mice. Splenocytes from d +3GFP+ DCreg-treated mice were stained for H-2Kb, H-2Kd, CD11c, and CL II for identification of DC subsets. Donor DC are H-2K d+CD11c+CL II+; Recipient DC are H-2K b+GFP-CD11c+CL II+; transferred DCreg are H-2K b+GFP+. The relatively large CL II+ CD11c- population in the donor gate is likely composed of activated T and B cells. (TIF) [file pone.0075158.s004.tif]

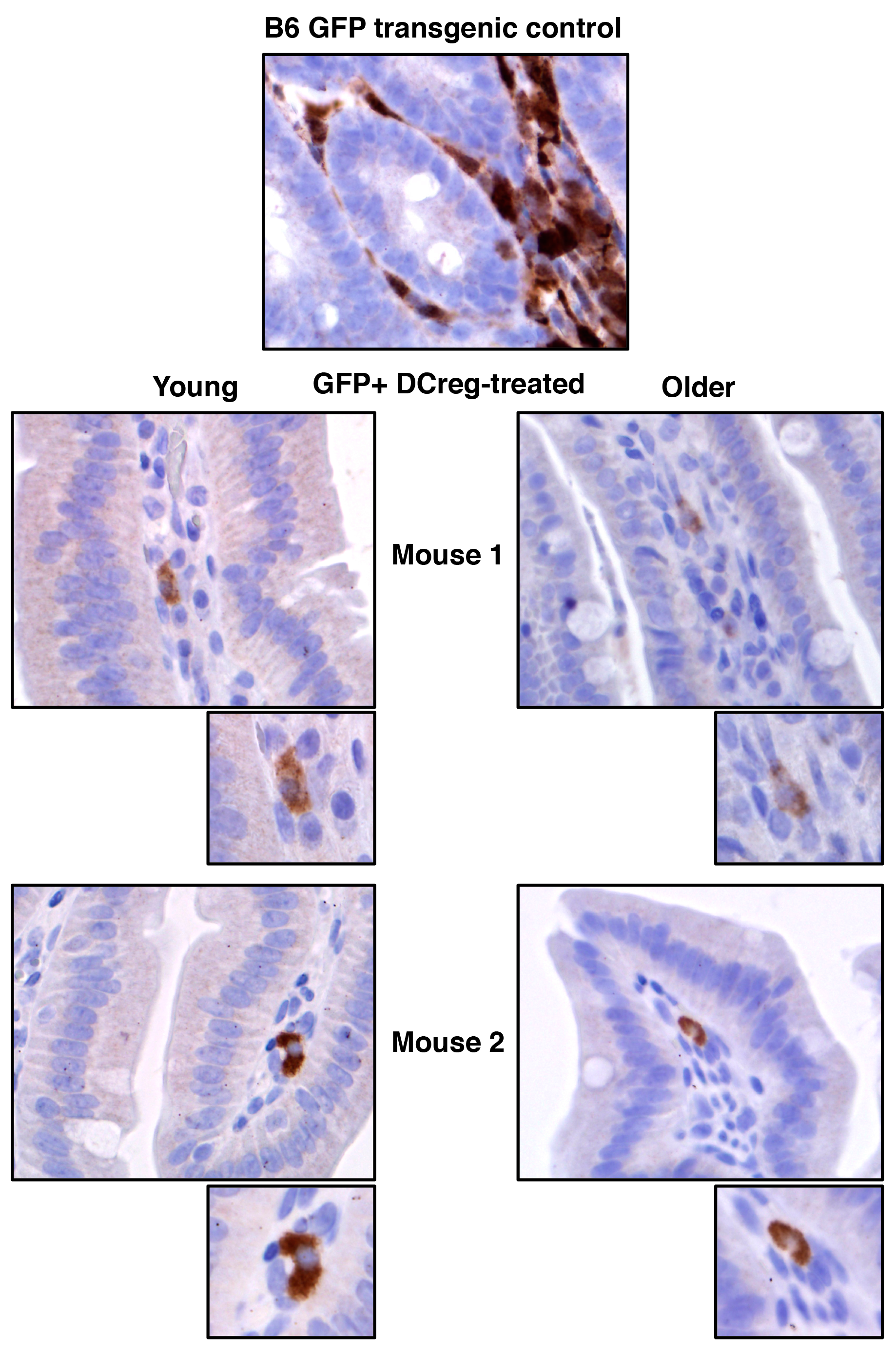

Supplement: Figure S5 — DCreg traffic to the small intestine, a GVHD target organ, following adoptive transfer. Young and older B6 mice were treated as described in Figure 3, except GFP+ DCreg were utilized for adoptive transfer. Following euthanasia, small intestine was prosected, rolled into a coil, and fixed, processed and sectioned as described in Materials and Methods. Sections were stained with a rabbit polyclonal anti-GFP antibody (Rockland Immunochemicals Inc., Gilbertsville, PA or Abcam, Cambridge, MA). Small intestine from GFP+ DCreg-treated BMT mice and untreated small intestine from GFP transgenic mice were stained for positive controls (top photograph). DCreg were also identified in the spleens of both young and older DCreg-treated BMT mice at both d +3 and d +5 (positive control concordant with flow cytometric results; data not shown). Negative controls included tissues obtained from naïve B6 mice or BMT mice that did not receive GFP+ DCreg as well as intestinal tissues obtained from GFP transgenic mice stained with isotype control antibody alone (data not shown). Original magnification = 40X and 60X in the upper and lower panels respectively, in the pair of images from each mouse. (TIF) [file pone.0075158.s005.tif]

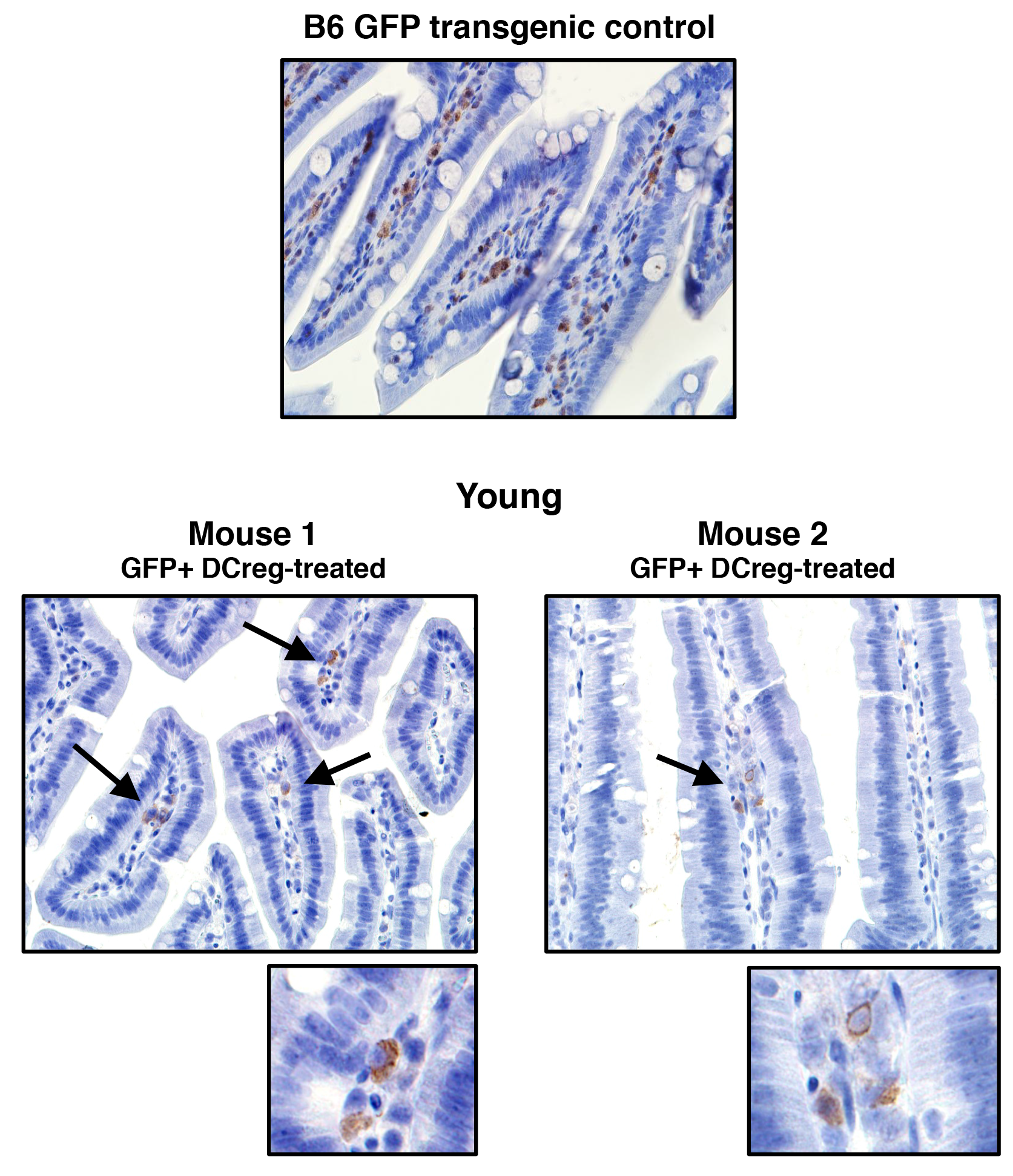

Supplement: Figure S6 — DCreg traffic to the colon, a GVHD target organ, following adoptive transfer. Young and older B6 mice were treated as described in Figure 3, except GFP+ DCreg were utilized for adoptive transfer. Following euthanasia, small intestine and colon were prosected, rolled into a coil, and fixed, processed and sectioned as described in Materials and Methods. Sections were stained with a rabbit polyclonal anti-GFP antibody (Rockland Immunochemicals Inc., Gilbertsville, PA or Abcam, Cambridge, MA). Colon from GFP+ DCreg-treated BMT mice and untreated colon from GFP transgenic mice were stained for positive controls (top photograph). DCreg were also identified in the spleens of both young and older DCreg-treated BMT mice at both d +3 and d +5 (positive control concordant with flow cytometric results; data not shown). Negative controls included tissues obtained from naïve B6 mice or BMT mice that did not receive GFP+ DCreg as well as intestinal tissues obtained from GFP transgenic mice stained with isotype control antibody alone (data not shown). Original magnification = 40X and 60X in the upper and lower panels respectively, in the pair of images from each mouse. (TIF) [file pone.0075158.s006.tif]
